# Protein-Losing Enteropathy as the First Presentation of Systemic Lupus Erythematosus in a Resource-Limited Setting in Sri Lanka: A Case Report

**DOI:** 10.7759/cureus.36619

**Published:** 2023-03-24

**Authors:** Ramanathan Ramesh, Navaneethakrishnan Suganthan, Gowry Selvaratnam, Uthayakumar Anushanth, Vadivel Vijitharan

**Affiliations:** 1 Medicine, Teaching Hospital-Batticaloa, Batticaloa, LKA; 2 Medicine, University of Jaffna, Jaffna, LKA; 3 Medicine, University Medical Unit, Teaching Hospital-Jaffna, Jaffna, LKA; 4 Gastroenterology and Hepatology, Teaching Hospital-Batticaloa, Batticaloa, LKA

**Keywords:** leucopenia, leg edema, hypoalbuminemia, enteropathy, sle

## Abstract

Protein-losing enteropathy (PLE) is one of the rare gastrointestinal manifestations of systemic lupus erythematosus (SLE), which can manifest several years before the diagnosis of SLE. PLE should be suspected in patients with hypoalbuminemia in the absence of urinary protein loss and normal liver functions without any other manifestations of malnutrition. Due to the non-specificity of the imaging and histological findings, it is difficult to diagnose PLE in resource-limited settings. Thus, it is underdiagnosed. We report the case of a 38-year-old Sri Lankan (South Asian) female who is a diagnosed patient with hypothyroidism and has presented with worsening generalized body swelling and ascites for two months. She had hypoalbuminemia without proteinuria. Thus, the clinical diagnosis of PLE was suspected. The diagnosis of SLE was suspected because of significant alopecia, high titer (1:1000) antinuclear antibody (ANA) positivity, and hypocomplementemia. Though confirmatory tests such as Tc-99 albumin scintigraphy and stool alpha-1 anti-trypsin were not available in our resource-limited setting, the diagnosis of the SLE-associated protein-losing enteropathy was made as the patient fulfilled the European Alliance of Associations for Rheumatology (EULAR) criteria for SLE and also by excluding all the other possible causes of PLE.

## Introduction

Systemic lupus erythematosus (SLE) is a multi-system inflammatory disorder that is more common in females of childbearing age. It is an autoimmune disorder marked by the presence of antibodies against nuclear and cytoplasmic antigens. Gastroenterological manifestations are noted in 40%-60% of SLE patients. The majority of the patients have mild symptoms, and life-threatening complications are rare. Common gastroenterological manifestations are pancreatitis, protein-losing enteropathy, acalculous cholecystitis, vasculitis, diarrhea, and ascites [1,2].

Protein-losing enteropathy (PLE) is a clinical condition in which there is shedding of protein through the gastrointestinal mucosa, which results in hypoalbuminemia and may manifest as generalized edema, ascites, and diarrhea [3]. Usually, the clinical diagnosis of PLE is suspected in patients with albumin<3.0g/L (the normal range is 3.4g/L - 5.4g/L) and normal urinary protein excretion. And PLE is a diagnosis of exclusion, and the protein loss through the GI tract can be confirmed by Tc-99m albumin scintigraphy and increased alpha 1- antitrypsin clearance in the stool [4]. Imaging might only show non-specific changes, such as thickened bowel, and a biopsy from the gastric or duodenal mucosa might show chronic non-specific inflammation of the lamina propria and ulcers [4].

A high index of suspicion is needed to diagnose PLE in SLE, as the majority of the investigations will be normal or non-specific. Thus, the literature regarding protein-losing enteropathy in SLE is not abundant, and except for the two large series by MoK et al. and XuanZhange et al., most of the others are isolated case reports or small-scale case series [5].

Protein-losing enteropathy was reported in several case reports to be diagnosed at the time of an SLE flareup, during advanced SLE, or even as the first presentation of SLE. PLE, as the first presentation of SLE, is rarely reported, and thus, we present a young female who presented with PLE as the first manifestation of SLE.

## Case presentation

A 38-year-old woman was diagnosed patient with well-controlled hypothyroidism and presented with a two-month history of worsening generalized body swelling that started from the ankles and gradually progressed to involve the abdomen and peri-orbital region. There was no diurnal variation in the symptoms of swelling in the body. She also complained of mild exertional dyspnea but denied orthopnea or paroxysmal nocturnal dyspnea. She refuses to experience chest pain, fatigue, or lethargy. She had a normal urine output, and there is no history of hematuria or frothy urine. No history of jaundice, melena, or rectal bleeding. She is clinically euthyroid and has no evidence of hypothyroidism, such as cold intolerance, hoarseness of voice, or menstrual abnormalities.

The patient reported one episode of symmetrical inflammatory-type small joint pain mainly involving the bilateral proximal interphalangeal joints and metacarpophalangeal joints, wrist joints, and knee joints without significant morning stiffness, which lasted a week and was treated as Rheumatic fever 18 years ago. The patient has significant hair loss and constitutional symptoms, but there is no history of photosensitivity rashes or malar rash. There is no history of miscarriages or sudden onset upper- or lower-limb weaknesses. There is no history of seizures or episodes of psychosis in the past. There is no history of Raynaud’s phenomenon, dry eyes, or redness of the eyes. There is no history of any autoimmune disorders or malignancies in her family.

Examination revealed generalized edema with ascites and bilateral pitting ankle edema. The patient was not pale or icteric. No photosensitivity rashes or malar rashes were observed. The patient had no oral ulcers or palpable lymph nodes. No goiter or organomegaly was noted. Her pulse rate was 90 bpm, and her blood pressure was 140/90 mmHg. Auscultation of the heart revealed a dual rhythm and no murmurs. Her jugular venous pressure was not elevated. The respiratory system examination was normal, with bilaterally equal vesicular breath sounds and no added breath sounds. Percussion over the lung zones was normal. Neurological examinations, including upper and lower limbs and cranial nerve examinations, were normal. Examination of her joints revealed no obvious joint swelling, tenderness or joint deformity.

Laboratory investigations revealed Hemoglobin of 12.4g/dl, Platelet of 220,000 per microliter, White Blood Cell (WBC) of 4100 per microliter, C-Reactive Protein (CRP) was 1.7 mg/L, and Erythrocyte Sedimentation Rate (ESR) was 6 mm/Hr. Her serum creatinine was 58 micromoles/L. Her Alanine transaminase (ALT) and Aspartate aminotrnsferase (AST) were normal. Her serum albumin was low (30g/dl) with a total protein of 68g/dl. Urine analysis reveals the absence of proteinuria and hematuria. Thus, a clinical diagnosis of protein-losing enteropathy was made, as there was hypoalbuminemia without proteinuria and otherwise normal liver function.

**Figure 1:**
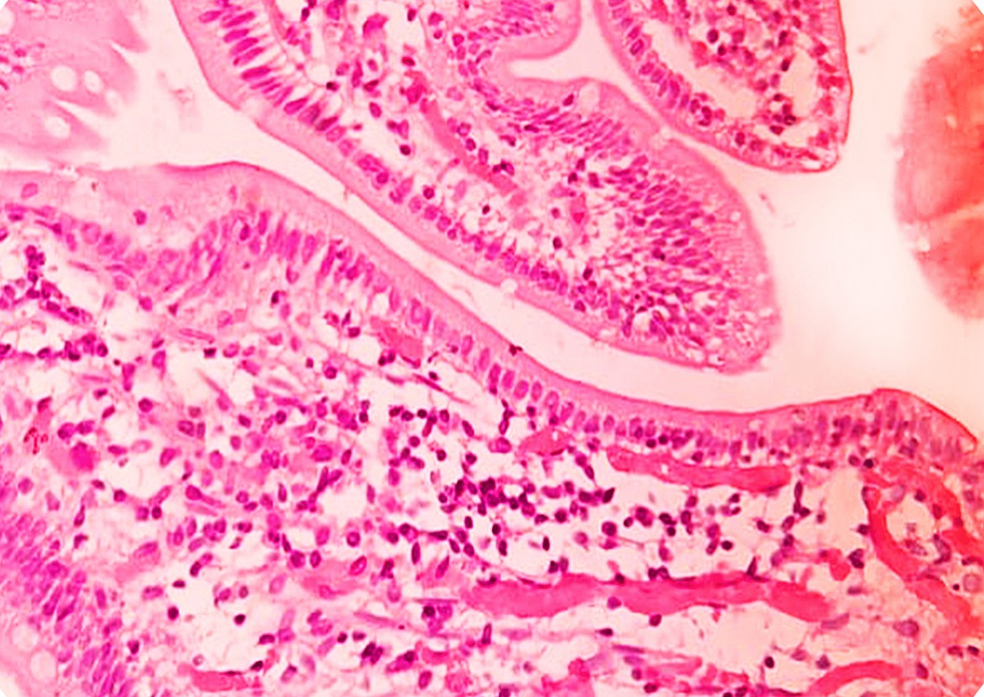
A biopsy from the terminal ileum reveals small intestinal mucosa with hyperplastic lymphoid follicles. Neither the features of IBD nor vasculitis were present.

To identify the cause of protein-losing enteropathy, the following investigations were done: An ultrasound scan of the abdomen revealed only moderate free fluid and no other obvious abnormalities. The contrast-enhanced computer tomography (CECT) of the abdomen was normal, and there was no mass lesion or lymphadenopathy. Also, there is no evidence to suggest bowel ischemia or small intestine strictures. Upper gastrointestinal endoscopy revealed a diffusely erythematous stomach, and a biopsy was taken from the mucosa of the stomach. And the gastric biopsies revealed gastric non-specific mucosa within normal limits. Colonoscopy was done, and the terminal ileal mucosa showed a granular appearance and shortened villi, but no terminal ileal ulceration was noted. Multiple biopsies were taken from the terminal ileum, and the histology revealed small intestinal mucosa with hyperplastic lymphoid follicles and showed neither the features of inflammatory bowel disease nor vasculitis was present (Figure 1).

As the patient had clinical features to suspect SLE, a series of investigations were launched to diagnose SLE. Her anti-nuclear antibody (ANA) was positive with a 1/1000 titer (Nucleolar pattern), but her anti-double-stranded DNA (ds-DNA) was negative. Both complement-3 (C3) and complement-4 (C4) levels were low (39mg/dl and 10mg/dl, respectively). The anti-cardiolipin antibody, lupus anticoagulant, and anti-beta 2 glycoprotein antibody were negative.

Her two-dimensional echocardiography (2D ECHO) revealed a 60% ejection fraction with no evidence of valvular disease or pericarditis. And, because the patient's clinical findings and investigations met the European Alliance of Associations for Rheumatology (EULAR) criteria-2019 with a score of 14 and fulfillment of at least one clinical criterion, SLE was diagnosed.

And certain other investigations were done to rule out the other common causes of protein-losing enteropathy. Anti-tissue transglutaminase antibody was negative (3.2AU/ml). Retroviral screening, Human Immunodeficiency Virus (HIV) 1, and HIV 2 were negative. And Mantoux’s test was negative. Her cytomegalovirus (CMV) antibodies, both immunoglobulin M (IgM) and immunoglobulin G (IgG), were negative. The stool full report revealed no parasitic infection. Serum calcium was normal, and carcinoembryonic antigen (CEA) and 5-hydroxyindolaceticacid (5-HIAA) in urine were negative. Her fasting blood sugar levels and thyroid function tests were both normal. Also, her anti-thyroid peroxidase antibody (anti-TPO antibody) was negative.

After excluding other possible causes, the diagnosis of “lupus-associated protein-losing enteropathy” was made. We have started oral prednisolone 1mg/kg body weight and hydroxychloroquine 200mg daily for her, and she has shown significant improvement in her body swelling, diarrhea, and serum albumin (40g/dl) in two weeks.

## Discussion

This case suggests that diagnosing protein-losing enteropathy in SLE needs a high level of clinical suspicion and could be the first presentation of SLE. Even though gastrointestinal manifestations are common in SLE, lupus-associated protein-losing enteropathy is rare.

Protein-losing enteropathy is usually suspected when a patient has clinical features of hypoalbuminemia (Serum Albumin<30g/dl) without significant urinary protein loss. Simultaneous detection of hypoalbuminemia and a 24-hour urinary protein less than 0.8g/24 hours has a sensitivity of 0.818 and a specificity of 0.989. And investigations such as Tc-99 albumin scintigraphy and stool alpha-1 anti-trypsin clearance can confirm the shedding of protein through the gastrointestinal tract [1].

The majority of the invasive tests, including tissue biopsies from gastro-intestinal mucosa and radiological investigations such as CECT abdomen, will reveal non-specific changes, thus making a diagnosis not easy. Due to the diagnostic difficulties and the rarity of the condition, only around 60 cases of lupus-associated protein-losing enteropathy were reported in the literature until 2010. And the majority of the cases reported are in the Asian population. According to the Mok group, the point prevalence of PLE in SLE is 3.2%. Lupus-associated PLE is more common in Asians, according to a systemic review by AL-Mogairen SM et al. [4]. The higher prevalence of SLE-associated PLE in the Asian population may be due to genetic and environmental factors. According to Tian XP et al., nearly half of all cases of PLE were the initial manifestation of SLE, but Gornisiewicz et al. reported PLE as a late presentation of severe SLE. 

Though our patient didn’t have a history of diarrhea, nearly 40% of reported patients had diarrhea. Severe hypoalbuminemia, severe hypocomplementemia, and hypercholesterolemia are commonly reported. The mechanism by which SLE causes PLE is yet to be known. Many hypotheses were proposed, including the increased vascular permeability of proteins caused by mesenteric vasculitis or vasodilation, vascular epithelial damage due to cytokines or complement, and lymphangiectasia [6]. 

Though colonoscopy is not diagnostic, Mogairen SM et al. reported non-specific mucosal thickening of the gastrointestinal tract in 44% of patients [4]. But tissue biopsy and histology may reveal lymphatic infiltration, mucosal edema, or lymphangiectasia in up to 80% of cases [4]. A non-invasive test called Tc-99 albumin scintigraphy (where the Tc-labeled human albumin is injected intravenously and then serial imaging of the abdomen is done) is the diagnostic test to confirm the protein shedding through the GI tract [4], though we couldn’t do it because of the unavailability of the test in our resource-limited setup and due to the financial restraints. Twenty-four-hour stool clearance of alpha-1 antitrypsin is an alternative diagnostic test, which is also not available in our center. As SLE-associated PLE is a diagnosis of exclusion, these tests are not essential for the diagnosis, especially in a resource-limited setting such as ours. The common causes of protein-losing enteropathies, such as coeliac disease, small intestinal bacterial overgrowth, tropical sprue, congestive heart failure, amyloidosis, Menetrier’s disease, and intestinal lymphangiectasia, should be excluded before making a diagnosis of lupus-associated protein-losing enteropathy.

In our patient, we excluded all the other possible causes for hypoalbuminemia, such as malabsorption, coeliac disease, lymphoma, heart failure, ulcerative colitis, Crohn’s disease, and amyloidosis through history, examination, and whenever necessary through appropriate investigations. And colonoscopic biopsies revealed no evidence of malabsorptive syndromes or inflammatory bowel disease, or bowel malignancies, but there is a nonspecific mucosal thickening that is reported in 44% of lupus-associated protein-losing enteropathy. The CECT abdomen revealed no evidence of lymphoma or lymphatic obstruction. 2D ECHO was normal, and there are no features of heart failure. The absence of albumin or microalbumin in urine ruled out renal albumin loss. The absence of parasites and fat globules in the stool full report safely excluded the parasitic infections and steatorrhea, respectively. The small intestinal fluid culture was negative and excluded small intestinal bacterial overgrowth. Retroviral studies were done, and HIV was excluded. Anti-tissue transglutaminase antibody was negative for coeliac disease. And also, the supportive evidence for the diagnosis of SLE, such as the highly positive antinuclear antibody (ANA) timer of 1:1000 and the fulfillment of the EULAR 2019 criteria, along with the exclusion of other possible causes, led to the diagnosis of lupus-associated PLE in our patient.

Corticosteroids and other immunosuppressants are used to treat SLE-associated PLE. And the majority of the patients with SLE showed an excellent response to corticosteroids [3]. In patients who are not responsive to corticosteroids, additional immunosuppressants such as Azathioprine, Cyclophosphamide, Mycophenolate, Methotrexate, and rituximab have shown benefit in certain case reports [1]. Lupus-associated PLE has an excellent prognosis (88% respond completely to corticosteroids), and very rarely mortality is reported [5].

## Conclusions

SLE-associated PLE is rare, and it is a diagnosis of exclusion. Asians have a higher point prevalence of SLE-associated PLE. Literature with regards to SLE-associated PLE is not abundant because of the rarity of the condition and also because of the difficulty in diagnosing the condition due to the non-specific findings in the imaging and histology. A high level of clinical suspicion is needed to diagnose PLE, as it may be the first presentation of SLE.
